# Evaluation of the Synergistic Effect of Graphene Oxide Sheets and Co_3_O_4_ Wrapped with Vertically Aligned Arrays of Poly (Aniline-Co-Melamine) Nanofibers for Energy Storage Applications

**DOI:** 10.3390/polym14132685

**Published:** 2022-06-30

**Authors:** Ishtiaq Ahmed, S. Wageh, Wajid Rehman, Javed Iqbal, Sadullah Mir, Ahmed Al-Ghamdi, Mohammad Khalid, Arshid Numan

**Affiliations:** 1 Department of Chemistry, Hazara University Mansehra, Mansehra 21300, Pakistan; ishtiaqmalik9996@gmail.com; 2 Department of Physics, Faculty of Science, King Abdulaziz University, Jeddah 21589, Saudi Arabia; wswelm@kau.edu.sa (S.W.); agamdi@kau.edu.sa (A.A.-G.); 3 Center of Nanotechnology, King Abdulaziz University, Jeddah 21589, Saudi Arabia; iqbaljavedch@gmail.com; 4 Department of Chemistry, COMSATS University Islamabad, Abbottabad Campus, Islamabad 45550, Pakistan; 5 Graphene & Advanced 2D Materials Research Group (GAMRG), School of Engineering & Technology, Sunway University, Bandar Sunway, Petaling Jaya 47500, Malaysia; khalids@sunway.edu.my

**Keywords:** poly(aniline-co-melamine), graphene oxide, cobalt oxide, in situ polymerization, ternary composites, energy storage, electrode, electrochemical performance, synergistic effect

## Abstract

In the present study, Co_3_O_4_ and graphene oxide (GO) are used as reinforcement materials in a copolymer matrix of poly(aniline-co-melamine) to synthesize ternary composites. The nanocomposite was prepared by oxidative in-situ polymerization and used as an electrode material for energy storage. The SEM images revealed the vertically aligned arrays of copolymer nanofibers, which entirely wrapped the GO sheets and Co_3_O_4_ nanoparticles. The EDX and mapping analysis confirmed the elemental composition and uniform distribution in the composite. The XRD patterns unveiled composites’ phase purity and crystallinity through characteristic peaks appearing at their respective 2θ values in the XRD spectrum. The FTIR spectrums endorse the successful synthesis of composites, whereas TGA analysis revealed the higher thermal stability of composites. The cyclic voltammetry, galvanostatic charge–discharge, and electrochemical impedance spectroscopy are employed to elucidate the electrochemical features of electrodes. The ternary composite PMCoG-2 displayed the highest specific capacity of 134.36 C/g with 6 phr of GO, whereas PMCoG-1 and PMCoG-3 exhibited the specific capacities of 100.63 and 118.4 C/g having 3 phr and 12 phr GO at a scan rate of 0.003 V/s, respectively. The best electrochemical performance of PMCoG-2 is credited to the synergistic effect of constituents of the composite material.

## 1. Introduction

So far, numerous renewable energy resources have been identified and practised to harvest energy. However, the intermittent availability of energy from renewable resources has averted the researchers’ attention to developing energy storage devices with mesmerizing energy storage capability, eco-friendly nature, low cost, fast charge—discharge mechanism, durability, and high power as well as energy density [1,2,3,4,5,6,7,8]. Batteries and supercapacitors have been utilized for energy storage applications. The energy storage mechanism in batteries is heavily dependent on the Faradaic reactions at their electrode and displays high energy density, but poor power density and slow charging impede its application, where high power density is required. However, supercapacitors have been the most alluring energy storage devices due to their speedy charge mechanism, long life, low cost, ecofriendly nature, and outstanding power density [3,9,10]. Although a supercapacitor displays superior attributes to batteries, it still needs to undergo sufficient improvement to circumvent its lower energy density [11,12,13].

The charge storage phenomena categorize the supercapacitors into the electrical double layer (EDLC) and pseudocapacitors. In EDLC, charge storage is based on the physical separation of charges at the electrode and electrolyte interface. In contrast, charge storage in pseudocapacitors occurs due to the fast reversible faradaic reactions [14,15,16,17,18]. Carbonaceous materials (graphite, activated carbon, carbon nanotubes, graphene, graphene oxide) are used to fabricate EDLCs electrodes. Due to the physical charge storage mechanism, EDLC displays good rate capability and long cyclic stability but suffers from poor energy density and specific capacitance [17,19,20]. On the other hand, pseudocapacitors show higher specific capacitance and cyclic stability than EDLCs due to Faradaic reactions [5,18]. The most appealing and popular materials for pseudocapacitors are conducting polymers such as PANI and metal oxides (RuO_2_, Mn_3_O_4_, SnO_2_, V_2_O_5_, Fe_2_O_3_, MnO_2_, Nb_2_O_5_ etc.) [10,18,21,22,23,24,25,26].

Among metal oxides, cobalt oxide (Co_3_O_4_) is the most promising contestant for supercapacitors electrode owing to its low cost, better performance, higher theoretical capacity, stable nature, ease of availability, environmental compatibility, and different oxidation states [5,18,26,27]. Unluckily, Co_3_O_4_ has showed poor electronic conductivity, which impedes ionic and electronic transport leading to poor energy density. The theoretical and experimental capacity disagreement is accredited to the abovementioned facts [26]. Multidimensional strategies such as the rational design of morphology, increasing surface area, doping with highly conductive materials such as rGO, CNTs, and conducting polymers were implemented to improve electrochemical performance significantly. Further, the use of different binding materials for electrode preparation also increases the impedance due to the increase in the dead volume of the electrode material [5]. Graphene is a promising candidate for electrode material owing to its high chemical stability, good conductivity, excellent mechanical strength, and high surface area. However, it is not easy to use the full potential of graphene for charge storage due to the restacking of graphene sheets, which reduces its surface area [28,29]. In order to avoid this issue, the surface of graphene is functionalized (graphene oxide) with epoxy, carbonyl, and hydroxyl functional groups, which not only resist the restacking of graphene sheets but also furnish its anchoring sites for the growth of the copolymer backbone as well as with nanoparticles [26,30]. The low conductivity and high particle aggregation of Co_3_O_4_ can be eliminated by combining graphene oxide with Co_3_O_4_ nanoparticles. The functional group sites can help to grow the Co_3_O_4_ nanoparticles, while Co_3_O_4_ nanoparticles, on the other hand, can act as a spacer between graphene sheets to avoid their restacking.

Polyaniline (PANI) has been identified as the most popular and ideal conducting polymer among the CPs due to its high theoretical capacitance, simple synthesis, low cost, and high thermal and electrochemical stability [8,26,30,31,32,33]. However, the pristine PANI is considered inferior electrode material in terms of low cyclic stability due to its deterioration when subjected to excessive charge—discharge cycles [34,35]. The use of different carbonaceous materials such as CNT, and rGO with metal oxide has been reported to improve the stability of PANI [36]. The synthesis of binary and ternary composites with carbonaceous materials with metal oxides and conducting polymers (CPs) is another approach to significantly improve the performance of the electrode material [7,37,38].

Based on the above discussion, a novel conductive copolymer matrix of poly(aniline-co-melamine) reinforced with Co_3_O_4_ and GO sheets as nanofillers is used to synthesize composites and explore their potential for energy storage applications. To the best of our knowledge, there is no report available on synthesizing graphene oxide and Co_3_O_4_ wrapped with vertically aligned arrays of poly (aniline-co-melamine) nanofibers composites for electrochemical energy storage application. The primary objective is to explore the synergy of copolymer matrix with different concentrations of GO sheets in ternary composites compared to its counterparts. The characterizations of ternary composites are conducted by applying various analytical techniques like XRD, SEM, EDX, EDX-mapping, FTIR, and TGA. The electrochemical performance is evaluated by cyclic voltammetry (CV), galvanostatic charge—discharge (GCD), and electrochemical impedance spectroscopy (EIS), respectively.

## 2. Materials and Methods

### 2.1. Materials

Graphite with 99% purity, measuring particles size <45 µm, was purchased from Sigma Aldrich to synthesize graphene oxide. Cobalt chloride (CoCl_2_·6H_2_O), ammonium persulphate (APS), sodium nitrate (NaNO_3),_ potassium permanganate (KMnO_4_), hydrogen peroxide (H_2_O_2_), aniline, and melamine were procured from DaeJung Korea. The aniline monomer was purified through vacuum distillation twice to eradicate the impurities and was stored in the dark. Acetone (99%), ethanol (99.8%), and methanol (99.8%) were obtained from Merck (Malaysia). During the synthesis of composites, deionized water (DI) was used.

### 2.2. Modified Hummer’s Approach for the Synthesis of Graphene Oxide (GO)

The modified hummer’s method was used for the synthesis of (GO). Initially, 2 g of graphite with particles size <45 µm and 2 g of NaNO_3_ were uniformly mixed in 90 mL of H_2_SO_4_ (98%) at 0–5 °C in an ice bath with constant stirring for 4 h. After complete homogeneity, 12 g of potassium permanganate was added slowly in such a way as to keep the temperature up to 15 °C. Then the mixture was stirred for 2 more hours at 35 °C. Next, the mixture was refluxed for 15–20 min at 98 °C and stirred for 2 h at room temperature. Afterwards, 40 mL of H_2_O_2_ was added to the mixture, and then 200 mL of DI water was added for dilution and stirred the mixture for 1 h. Later, the mixture was left undisturbed for 4 h. Lastly, through centrifugation, the (GO) was thoroughly washed with 10% HCl and then with sufficient water to remove the impurities to obtain neutral pH. The resultant gel was dried for 6 h in a vacuum oven at 50 °C to obtain the powder of (GO) [39].

### 2.3. Synthesis of Co_3_O_4_ Nanoparticles

A simple co-precipitation method was used to prepare the Co_3_O_4_ nanoparticles. First, a 0.15 M CoCl_2_·6H_2_O solution (100 mL) was prepared in DI water by stirring for half an hour. Then, a 0.8 M NaOH (100 mL) was slowly added dropwise at ambient temperature at 500 rpm in the cobalt precursor solution. In the beginning, the pink color of cobalt precursor was turned into dark green and finally changed to dark brown. The resultant mixture was thoroughly stirred for about 1 h after adding NaOH solution. The synthesized nanoparticles were separated through centrifugation at 4000 rpm. The nanoparticles were washed with plenty of water and, finally, ethanol. The precipitate was dried at 110 °C for 24 h in an oven, followed by calcination at 300 °C for 3 h to obtain Co_3_O_4_ nanoparticles [40,41,42].

### 2.4. Synthesis of Ternary Composites of Copolymer/Co_3_O_4_/GO, Copolymer, PANI

**Figure 1:**
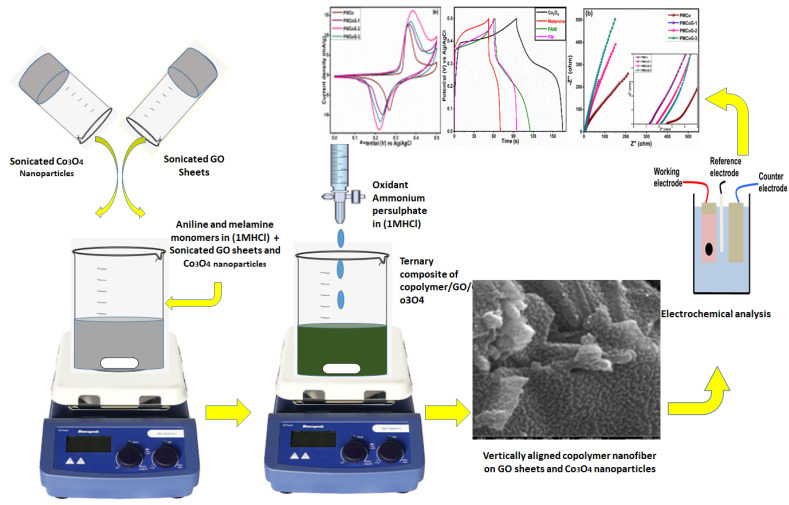
Schematic illustration of the synthesis of the composites.

A 0.0215 M solution of aniline monomer (15 mL) was prepared by homogenizing doubly distilled aniline in 1 M HCl under constant stirring. Later, 60 mg of melamine was added and stirred for half an hour. The (GO) and Co_3_O_4_ nanoparticles sonicated for 2 h in deionized water separately were added dropwise in the above mixture and stirred for 30 min. Subsequently, a dropwise addition of 17.5 mL of 0.0268 M ammonium persulphate as an oxidizing agent in 1 M HCl was carried out. The dark green color indicated the successful synthesis of the composite of the conductive copolymer. The mixture was stirred constantly for 3 h and left undisturbed for 12 h. The composite was then separated by filtration, washed with 0.5 M HCl (100 mL), and later with ample DI water until the filtrate became colourless The obtained residue was washed with ethanol and acetone. The washed composite was dried at 50 °C for 24 h. Figure 1 shows the schematic illustration of the synthesis of ternary composite. A similar procedure was followed for preparing different combinations of composites and pristine PANI as given in the Table 1 [43,44].

### 2.5. Characterization

The structure, composition, morphology, and homogeneity of pristine and copolymer composites materials were elucidated by FTIR, XRD, SEM, EDX, and mapping. The Perkin Elmer (Spectrum Two, ATR Sample base plate Diamond) FTIR spectrophotometer was employed to obtain the FTIR spectrum. The X-rays (JDX-3532, JEOL, Tokyo, Japan) diffraction analysis examined the structure and phase purity of pristine and composites at 2θ angles ranging from 5–80 °C. The morphology of the samples was revealed through SEM images at various magnifications (SEM, MIRA3 TESCAN). The elemental composition and homogeneous dispersion of components of the composites were determined by (SEM, MIRA3 TESCAN). Finally, the thermal stability of the samples was assessed by the thermogravimetric analysis performed by TGA Q500 within a temperature range of 0–800 °C.

### 2.6. Fabrication of Electrodes and Electrochemical Studies

A pre-cleaned nickel foam with an area of 1 × 1 cm^2^ has been employed to fabricate the electrode of pristine and composites. A homogeneous slurry of all the materials was prepared by intimate mixing of (75 wt%) of electrode material with acetylene black (15 wt%) and polyvinylidene fluoride (PVdF) (10 wt%) in NMP(N-Methyl-2-Pyrrolidone). The mixture was stirred for 12 h at ambient temperature to attain complete homogeneity. The electrode was prepared by carefully dispersing a drop of the electroactive material on the chemically cleaned nickel foam and dried in an oven at 90 °C. All electrodes were fabricated with a mass loading of ~5.00 ± 0.05 mg of the requisite electrode material on nickel foam. The electrochemical behavior of fabricated electrodes was examined by Gammry Interface 1000 Instrument, Warminster, PA, USA, electrochemical workstation. CV curves were recorded at a voltage of 0–0.5 V using Ag/AgCl as a referenced electrode and platinum wire as a counter electrode. GCD measurements were conducted at a potential of 0.0–0.5 V at various current densities ranging from 1 to 3 A/g. EIS was executed in a frequency range of 0.01–100 kHz at a fixed AC voltage of 10 mV (RMS). During the electrochemical studies, 0.1 M KOH served as an electrolyte.

## 3. Results and Discussion

### 3.1. SEM Analysis

**Figure 2:**
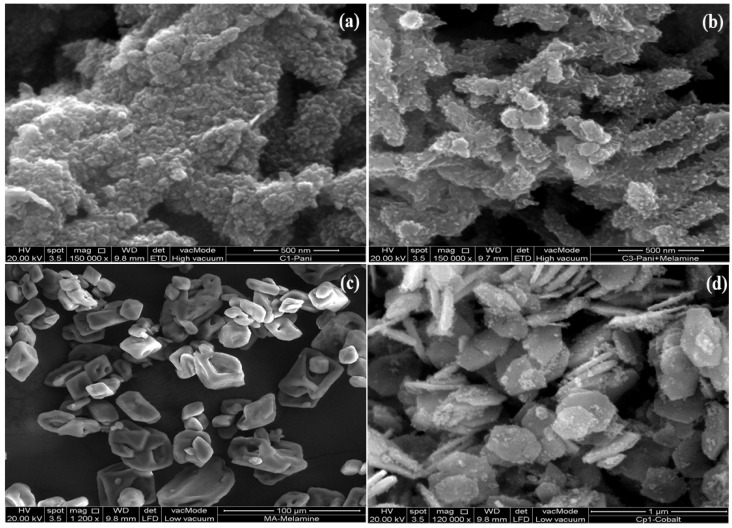
SEM images of (**a**) PANI (**b**) Copolymer (**c**) Melamine (**d**) Co_3_O_4_ nanoparticles.

Different magnifications of SEM have been applied to reveal the morphological features of the pristine and copolymer composites. Figure 2a shows that pristine PANI appeared as nanoparticles of various sizes ranging from 30 to 80 nm [45]. The nanoparticles of PANI combine to assume the shape of fibres, which mingle to form a fibrous network [46]. The poly(aniline-co-melamine) shows a slight change in morphology due to the incorporation of melamine monomers, as shown in Figure 2b. The particle size of the copolymer was found to be in the range of 20 to 40 nm. The nanoparticles of copolymer underwent aggregation to form the fibrous network. Figure 2c shows morphological features of melamine with irregular, hollow macroparticles of different sizes and shapes. The Co_3_O_4_ nanoparticles depicted hexagons of diverse diameters of 220 to 550 nm with widths ranging from 25 to 80 nm without agglomeration, as illustrated in Figure 2d.

**Figure 3:**
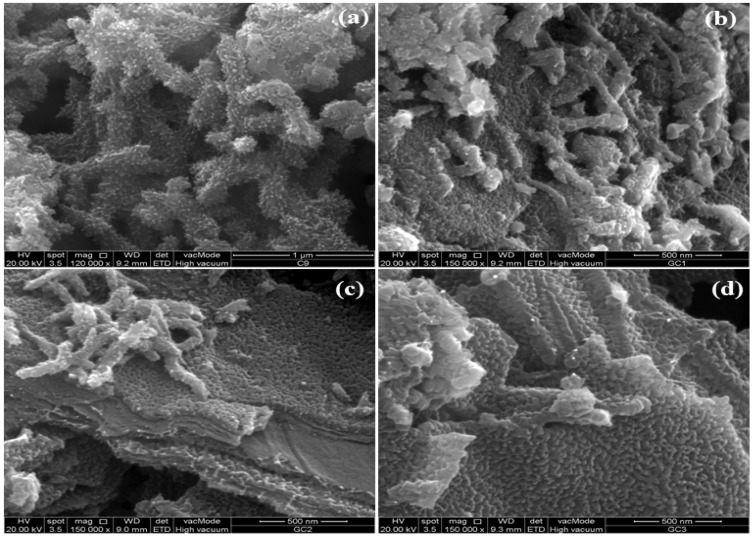
SEM images of composites (**a**) PMCo (**b**) PMCoG-1(**c**) PMCoG-2 (**d**) PMCoG-3.

The morphological aspects of copolymer composites are displayed in Figure 3a–d with varying concentrations of Co_3_O_4_ and GO. It is vivid from SEM images that GO sheets have been completely wrapped by the nanofibers of copolymer furnished by the Co_3_O_4_ nanoparticles. The nanofibers are arranged in vertical arrays on the GO sheets, preventing the restacking of GO sheets and enhancing the surface area. The basal plane and edges of GO are occupied with various oxygen-containing functional groups, which facilitates the attachment of copolymer on its surface [47]. The pristine GO shows its characteristic peaks in FTIR and XRD spectrum. However, these peaks completely disappeared in the composites, which may be due to the meager concentration of GO, or due to the complete wrapping of GO sheets with nanofibers of the copolymer. The copolymer nanofibers have also embedded the Co_3_O_4_ nanoparticles as vivid from the SEM images because no Co_3_O_4_ nanoparticles can be seen in the composites.

**Figure 4:**
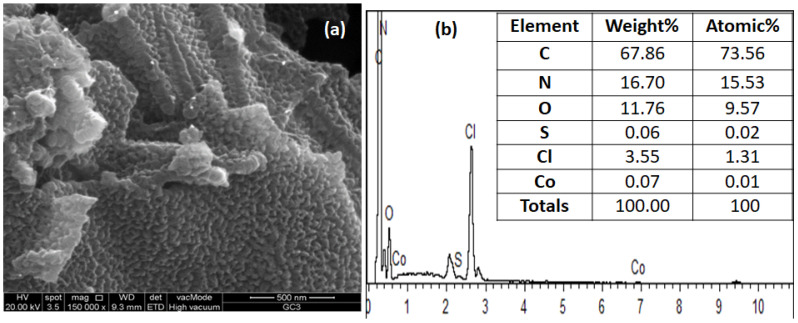
(**a**) SEM image of PMGCo-2, (**b**) EDX analysis showing the elemental composition of PMGCo-2 composite.

Figure 4 illustrates the elemental composition of composites. EDX examination shows that composites are composed of nitrogen, carbon, oxygen, cobalt, sulfur, and chlorine and endorse the purity of composites. The small concentration of sulphur and chlorine in the sample is due to the use of dopant HCl and ammonium persulphate as oxidizing agents [44]. The elemental composition of composites validates the successful synthesis of copolymer composites.

### 3.2. Fourier Transformed Infrared (FTIR) Studies

**Figure 5:**
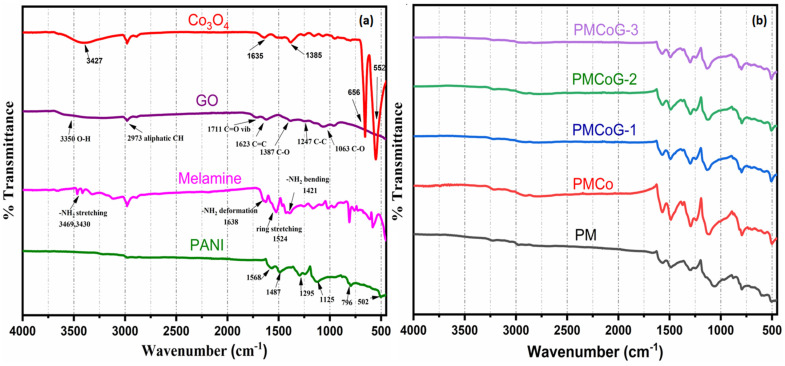
FTIR spectrum of (**a**) PANI, melamine, GO and Co_3_O_4_ (**b**) PMCo, PMCoG-1, PMCoG-2 and PMCoG-3.

Figure 5a,b illustrate the FTIR spectra of Co_3_O_4_ nanoparticles, PANI, GO, melamine, and copolymer composites ranging from 450–4000 cm^−1^. The Co_3_O_4_ nanoparticles showed characteristic peaks at 552 and 656 cm^−1^. The Co-O stretching vibrational mode is responsible for the 552 cm^−1^ IR band, whereas the appearance of IR band at 656 cm^−1^ is ascribed to O-Co-O bridging vibration due to Co-O linkage. The IR band at 1635 cm^−1^ is credited to the absorbed water and is attributed to the H-O-H bending vibrations [48,49]. The IR band at 3427 cm^−1^ is credited to O-H stretching vibration mode due to adsorbed water on the surface of Co_3_O_4_ nanoparticles [5,50]. The pristine PANI is characterized by the IR peaks index at 796, 1125, 1241, 1295, 1487, and 1568 cm^−1^ respectively. The out-of-plane and in-plane bending vibrational mode of C–H bond is responsible for the appearance of IR bands at 796 and 1125 cm^−1^. The stretching vibrations of C-N and C=N bond in PANI are credited to IR band at 1295 cm^−1^. The IR bands that occurred at 1125, 1241, and 1295 cm^−1^ are considered the characteristic IR peaks of pristine PANI [45,47,51].

The IR identification bands for melamine appeared at 3469 and 3430 cm^−1^. These IR bands are credited to the stretching vibrations of –NH_2_ bond, as demonstrated in Figure 5a. However, an IR band that appears at 1638 cm^−1^ is credited to the –NH_2_ deformation mode. The stretching of the melamine ring is responsible for the appearance of IR bands at 1524 and 1421 cm^−1^, respectively, whereas the IR band indexed at 1158 cm^−1^ is assigned to C-N stretching vibration. The characteristic IR band for azo (N=N) bonds at 1500 cm^−1^ could not be seen in the copolymer and the composites. It might be due to the meager concentration of melamine and the overlapping with IR bands of PANI [51].

FTIR spectra of composite (PMCo) reveal the small shifting of characteristic peaks of Co_3_O_4_ from 552 and 656 cm^−1^ to 576 and 637 cm^−1^, respectively. The peak shift is credited to van der Waal interactions between Co_3_O_4_ nanoparticles and copolymer chains [48]. An IR band that appears in the FTIR spectrum of GO indexed at 3350 cm^−1^ is due to the vibrational stretching mode of O-H bond (Figure 5a). The IR band at 2973 cm^−1^ is attributed to the stretching mode of C-H bonds, whereas the IR band of C=C stretching mode has appeared at 1623 cm^−1^ as shown in Figure 5a. The IR band at 1623 cm^−1^ is assigned to the stretching mode of C=C bonds, whereas the band at 2973 cm^−1^ is ascribed to the stretching mode of the C-H bond. The IR peaks at 1063, 1247, and 1711cm^−1^ are credited to stretching vibration of C-O in C-O-C in an epoxy group, C-OH, and stretching of C=O in the carboxylic group, respectively. The appearance of the above-mentioned peaks in the FTIR spectrum of GO, is sufficient evidence that GO is heavily occupied by the various oxygenated functional groups. These oxygenated groups are responsible for developing π-π and hydrogen bonding between copolymer chains and GO sheets [47,52].

The addition of 6 phr of melamine in the aniline monomer to synthesize poly (aniline-co-melamine) causes a slight shift of PANI band in the copolymer. Nevertheless, no profound effect is observed in the IR bands of PANI due to the small concentration of melamine as peaks of melamine are overlapped by the PANI (Figure 5b) [34,51]. A significant drop in the intensities of GO and Co_3_O_4_ nanoparticles is noticed in the FTIR spectra of copolymer /GO/Co_3_O_4_. This clearly shows that GO sheets and Co_3_O_4_ nanoparticles have been completely refuged by the fibres of the copolymer in the composites, as evident from SEM images.

### 3.3. XRD Analysis

**Figure 6:**
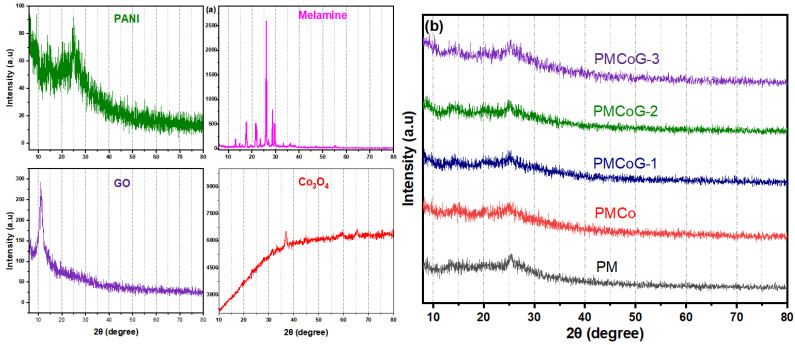
XRD spectrum of (**a**) PANI, GO, melamine and Co_3_O_4_ (**b**) PMCo, PMCoG-1, PMCoG-2 and PMCoG-3.

X-ray diffraction analysis examines the structural features and crystallinity of pristine PANI, Co_3_O_4_ nanoparticles, melamine, GO, copolymer and copolymer composites with different GO concentrations. The Co_3_O_4_ nanoparticles displayed its characteristic peaks indexed at 2θ values of 31.2°, 36.85°, 44.82°, 59.37° and 65.25°, corresponding to the lattice planes (220), (311), (400), (511) and (400), harmonized with cubic crystal system, showing space group FD3m (227) (Figure 6a). The Co_3_O_4_ nanoparticles showed harmonization with JSCPD No. 01-080-1532 as per the literature [38,53,54].

A sharp peak at 2θ value of 11.2° for GO corresponds to plane (001), as illustrated in Figure 6a. The attachment of different oxygen-containing functional groups at the basal plane and edges of GO plays a crucial role in enhancing its dispersibility. It also facilitates the anchoring of different organic and inorganic groups to form composites. Further, water molecules can be readily adsorbed on the surface of GO due to the presence of oxygenated functional groups, which is responsible for the more interplanar distance in GO sheets (0.87 nm) than graphite (0.340 nm) [10,39,45,47,55,56,57] Figure 6a. The pristine PANI is identified by the appearance of XRD peaks indexed at 25.21°, 21.07° and 15.2° synchronized with the semi-crystalline plane at (200), (020), and (011), respectively, validating the successful synthesis of polyaniline [7,47,56,58]. The peaks indexed at 21.07° and 25.21° at 2θ (Figure 6a) are due to the systematic occurrence of quinoid and benzenoid rings in the polymer backbone [48]. The characteristic peaks of GO have completely disappeared in the copolymer composites. The slight shifting of the 2θ values indexed at 24.84°, 20.04° and 14.47° is observed in the composite than in pristine PANI. The XRD peaks shift indicates π-π interaction between the polymer backbone and GO sheets [59].

Melamine is characterized by the appearance of peaks indexed at 2θ values of 17.63°, 21.69°, 26.15°, 28.73°, and 29.840, respectively. A slight shifting in the XRD peaks of PANI is ample evidence for the synthesis of copolymer due to the addition of 6 phr of melamine. However, the XRD pattern of PANI completely dominated the copolymer XRD pattern, which might be due to the lower concentration of melamine [51]. A slight shift is observed for Co_3_O_4_ nanoparticles in the copolymer, as illustrated in Figure 6b, due to the existence of van der Waal forces between Co_3_O_4_ and the backbone of the copolymer [48].

The composites of copolymer with GO showed that the characteristics peak of GO at 11.2° has been wiped out in their XRD pattern. The addition of different concentrations of GO and Co_3_O_4_ nanoparticles has no profound effect on the XRD spectrum of the copolymer; however, a slight shift in the XRD peaks of the copolymer is noticed. The arrays of copolymer fibres on GO sheets act as spacers and not only prevent its restacking but also enhance its surface area and, ultimately, the electrochemical performance.

### 3.4. Thermogravimetric Analysis

**Figure 7:**
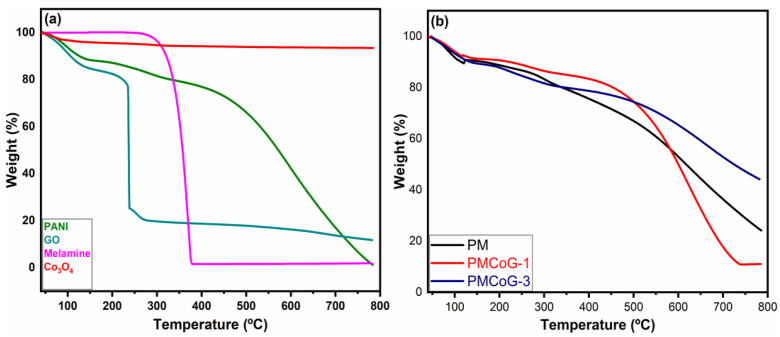
TGA analysis of (**a**) PANI, GO, Melamine and Co_3_O_4_ (**b**) PM, PMCoG-1 and PMCoG-3.

The thermal stability of pristine and composites was examined through thermogravimetric analysis. All the samples were heated within a temperature range of 30–800 °C in N_2_ atmosphere at a heating rate of 30 °C min^−1^, as illustrated in Figure 7a,b and Table 2. The Co_3_O_4_ nanoparticles displayed outstanding thermal stability and exhibited just a weight loss of about 6.55% throughout the whole temperature range within three stages. The first weight loss of 4.22% occurred up to 150 °C, related to the removal of adsorbed water in the nanoparticles (Figure 7a). A weight loss of 2.33% is observed up to 781 °C due to the decomposition of Co_3_O_4_ nanoparticles into Co_3_O_4_ and oxygen, as given below [43,60].

Co_3_O_4_ → 3CoO + 1/2O_2_

PANI homo-polymer showed weight loss in three stages (Figure 7a). The first weight loss of 11.62% occurred up to 150 °C, which is credited to the disintegration of untreated monomers and the removal of adsorbed water. The second weight loss of about 9.52% is accompanied by the removal of dopant [48] that is adhered to the PANI backbone within a temperature range of 150–360 °C. The significant and final weight loss of 77.63% occurred from 360–784 °C due to the breakdown of the backbone of the polymer matrix [46,48,61,62]. GO showed a first weight loss of 15.29% up to 150 °C due to the removal of adsorbed water. A significant weight loss of 73.6% occurred up to 360 °C because of the disintegration of the carbon skeleton of the GO sheets. The final weight loss (14.54%) is credited to eradicating thermally stable oxygenated functional groups attached to GO skeleton up to 784 °C. A total weight loss of 88.23% is observed regarding thermal changes of GO with a residue of 11.77% up to 784 °C [63,64,65,66].

In the beginning, melamine showed thermal stability up to 280 °C and displayed a weight loss of 56% up to 360 °C. However, it showed a 41.99% decrease in weight up to 782 °C with a total weight loss of 98.08% with 1.92% residue left at the end. The comparison of PANI and melamine thermal analysis (Figure 7a) reveal that PANI displayed gradual thermal changes and higher thermal stability than melamine.

The copolymer displayed greater stability to heat compared to pristine PANI. The higher thermal stability is due to the stronger interaction between the PANI and melamine backbone (Figure 7b) [51]. The copolymer displayed better thermal stability after 500 °C compared to PANI.

The composite PMCoG-1 displayed a weight loss of 8.76% up to 150 °C and is ascribed to the loss of water adsorbed in the composites. A second weight loss of 15.3% happened up to 360 °C, whereas the 3rd weight loss of 64.91% was noticed after the completion of thermal changes. The PMCoG-1 displayed an overall weight loss of 88.97% up to 800 °C. The composite PMCoG-3 exhibited remarkable thermal stability and showed a weight loss of 10.61% up to 150 °C. The composite showed a total weight loss of 55.86% at the completion of thermal changes with a residue of 44.14%. The composite PMCoG-3 displayed the highest thermal stability compared to other composites, ascribed to the formation of sound interaction because of the synergistic effect of components. It is observed that an increase in the concentration of GO from 3 phr to 12 phr has a profound effect on the thermal stability of the composites. The comparison of thermal changes of pristine and composites reveals the thermal stability order as Co_3_O_4_ > PMCoG-3 > PM > PMCoG-1 > PANI.

### 3.5. Electrochemical Study

**Figure 8:**
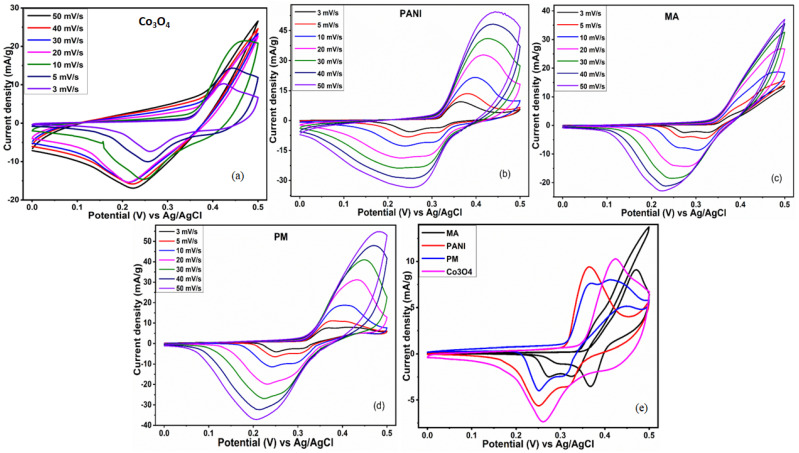
CV pattern of (**a**) Co_3_O_4_ (**b**) PANI (**c**) melamine (**d**) PM (**e**) Comparison of CVs.

**Figure 9:**
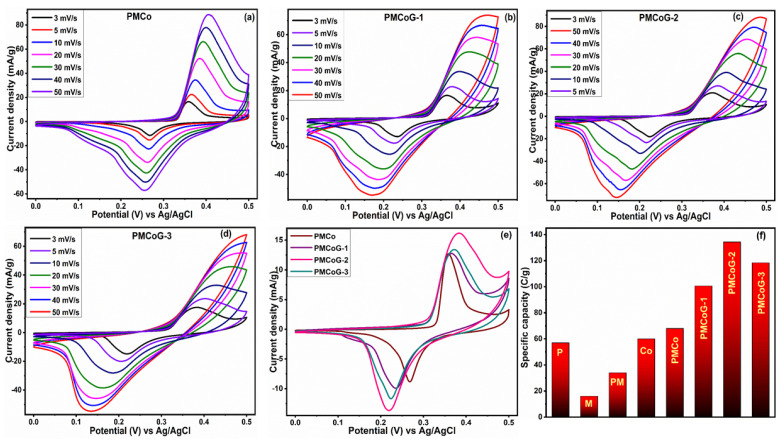
Displaying the CV pattern of (**a**) PMCo (**b**) PMCoG-1 (**c**) PMCoG-2 (**d**) PMCoG-3 (**e**) CVs comparison (**f**) comparison of specific capacity of controlled and copolymer composite.

A three-electrode system is used to evaluate the inherent electrochemical performance of pristine and composites electrodes in 1 M KOH (Figure 8 and Figure 9). CV curves for each electrode displayed redox peaks at diverse scan rates. The shape of the CV curve gives valuable information about the behavior of the electrode, which indicates the battery-grade attributes for each electrode [67]. The well-disciplined arrangements of the CV curves at different scan rates [38] validated their decent rate capability [55,68]. The harmonized CV curves at various scan rates also validate the pertinent nature of 1 M KOH as an electrolyte [69]. The EDLC attribute is anticipated on behalf of graphene sheets in the ternary composites, whereas the Co_3_O_4_ nanoparticles are expected to contribute to its battery behavior [56]. The peak-shaped CV of the electrode is an indicator of capacitive (due to GO) as well as diffusive behavior (due to Co_3_O_4_). The slight shift in the CV curve is attributed to the sluggish movement of the electrolytic ions to the electrode at high current densities [70].

The distortion observed in the CV curve of Co_3_O_4_ nanoparticles might be due to the interruption in the diffusion of electrolytic ions toward the electrode (Figure 8a). The pure PANI electrode (Figure 8b) displays a peak current density of 47 mA/g at 50 mV/s. The existence of PANI in different oxidation states, such as fully reduced leucoemeraldine, the fully oxidized Pernigraniline, and the half-oxidized emeraldine forms are liable for its redox behavior [71]. The PANI undergoes transitions from semiconducting lecuoemaraldine form into conducting polaronic emeraldine form and polaronic emeraldine into pernigraniline structures throughout redox reactions [72]. As illustrated in Figure 8c, melamine displays a current density of 37 mA/g at 50 mV/s; however, copolymer stands at 55 mA/g at 50 mV/s, as shown in (Figure 8d). The copolymer shows better electrochemical behavior in comparison to pristine components of composites. The improved electrochemical attributes of the copolymer are credited to adding 6 phr of melamine to the copolymer [34].

The composite PMCo displays a peak current density of 88.6 mA/g at 50 mV/s, as illustrated in Figure 9a. The composites PMCoG-1, PMCoG-2, and PMCoG-3 showed peak current densities of 73.87, 88.39, and 68.36 mA/g, respectively, when scanned at 50 mV/s as shown in Figure 9b–d. The composite PMCoG-2 displays better electrochemical behavior than its counterparts because of the synergistic association among the components. The comparison of CVs of composites is given in Figure 9e.

Compared to other composites, the highest area occupied by the CV curve of composite PMCoG-2 displayed the best electrochemical performance. The following expression (1) can be used to calculate the specific capacity of electrodes by using its CV curves

(1) $$\mathrm{Qs}=\frac{1}{mv\;}\overset{Vf}{\underset{Vi}{\int }}I\times VdV$$

In the above expression, Qs, *m*, and *v* are an integral part of the equation and represent the specific capacity (C/g), active mass (g), scan rate (mV/s), and area under the curve, respectively.

The Qs for melamine, PANI, Co_3_O_4_, and the copolymer is calculated to be 17.8 C/g, 53 C/g, 70 C/g, and 34.4 C/g, respectively. The Qs for composite PMCo and ternary composites PMCoG-1, PMCoG-2, and PMCoG-3 were found to be 68.73, 100.63, 134.36, and 118.4 C/g, respectively. Comparing the Qs of pristine and copolymer composites demonstrates that ternary composites display better electrochemical signatures than binary and pristine components. In composites, it is observed that GO concentration up to 6phr showed the best electrochemical behavior in synergistic association with Co_3_O_4_ nanoparticles and copolymer, which might be due to the better morphology of the composite. However, as the concentration of GO is increased from 6 phr to 12 phr, the electrochemical performance of the electrode has been dropped due to heaping of GO sheets and ultimately decreases the electrochemical sites.

### 3.6. Galvanostatic Charge—Discharge (GCD) Studies

**Figure 10:**
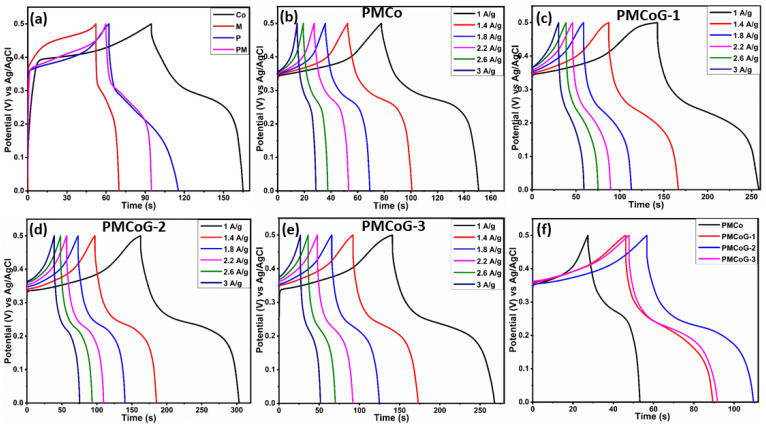
Showing the GCD curves (**a**) comparison of Co_3_O_4_, melamine, APNI and PM (**b**) PMCo (**c**) PMCoG-1 (**d**) PMCoG-2 (**e**) PMCoG-3 (**f**) GCDs comparison of composites.

**Figure 11:**
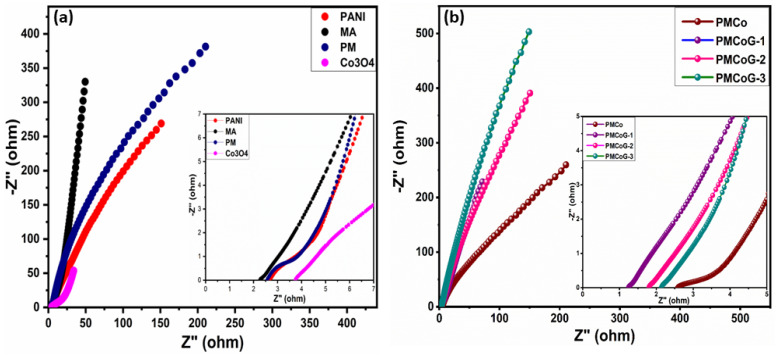
(**a**) EIS of PANI, melamine, PM, and Co_3_O_4_ (**b**) EIS of PMCo, PMCoG-1, PMcoG-2 and PMCoG-3.

The GCD is an old-fashioned technique but still good enough to evaluate the electrochemical performance in terms of power density, specific capacity, cyclic stability, and energy density of electrode material. The GCD was performed at a potential window of 0.0 to 0.5 V vs. Ag/AgCl standard electrode at a current density of 1–3 A/g, as shown in Figure 10 and Figure 11. The nonlinear GCD curves with little shoulder indicate faradaic behavior of the electrode [73,74]. An inverse relationship is observed for the Qs of the electrode with respect to current density. It shows that with the increase in current density, the electrolytic ions cannot obtain enough time to access the electroactive sites of the electrode and deteriorate their electrochemical performance [38,75]. The specific capacity can be calculated by employing the expression (2)

(2) $$\mathrm{Qs}=\frac{I\times \Delta t}{m}$$

where *I* represents the current in ampere, Δt and *m* show the discharge time and mass loading of the electrode material, respectively.

By using the expression (2), the Qs of melamine, Co_3_O_4_ nanoparticles, PANI, and copolymer were found to be 16, 60, 57, and 34 C/g at 1 A/g, respectively (Figure 10a). The Qs of composite PMCo turned out to be 72.9, whereas the composites PMCoG-1, PMCoG-2, and PMCoG-3 showed the Qs of 115, 139, and 126 C/g, respectively, as illustrated in Figure 10b–f.

The better charge—discharge attributes of ternary composites are credited to the synergistic association among the components. Comparing the Qs values of the composites reveals that PMCoG-2 showed the highest Qs. It is concluded that, as GO concentration was increased from 3 phr to 6 phr, the electrochemical performance is increased, which is ascribed to the availability of a more significant number of electroactive chemical sites. However, when the GO concentration was increased to 12 phr, a decline in electrochemical activity of the composite was noticed, which is due to heaping of GO sheets.

The vertical growth of copolymer nanofibers on GO sheets prevents their restacking. It also helps to prevent the aggregation of Co_3_O_4_ nanoparticles. However, GO concentration up to 12 phr deteriorates the electroactive chemical sites and ultimately drops the Qs value [38].

### 3.7. Electrochemical Impedance Spectroscopy (EIS) Studies

This technique develops a better understanding of intrinsic resistance, diffusion of ions, and charge transfer kinetics at the electrode [10]. In the low-frequency region, the appearance of a vertical line parallel to the imaginary axis relates to the capacitive signature of the electrode and is termed the Warburg effect [57,76]. The equivalent series resistance (ESR) of the electrode is credited to internal resistance offered by the electrode, resistance due to electrolytic ion and contact resistance [77], and is calculated from the first place where the semicircle touches the real impedance axis [10,38].

As illustrated in Figure 11a, the Rct for PANI, Co_3_O_4_, copolymer, and melamine were found to be 2.23, 15.4, 2.24, and 1.80 Ω, respectively. The melamine shows the least Rct value among the controlled samples and exhibits good conductive nature. The composite PMCo displays Rct value of 1.43, whereas the composites PMCoG-1, PMCoG-2, and PMCoG-3 showed Rct values of 0.54, 0.7 and 0.83 Ω, respectively (Figure 11b).

The ESR value for Co_3_O_4_ nanoparticles was calculated to be 4.13 Ω. However, melamine, PANI, and copolymer showed ERS values of 2.2, 2.27, and 2.26 Ω, respectively. Furthermore, the composites, PMCo, PMCoG-1, PMCoG-2, and PMCog-3, displayed ESR values of 2.57, 1.26, 1.86, and 2.17 Ω, respectively. The melamine showed the best capacitive behavior among the controlled samples as it displayed the highest slope parallel to the imaginary axis. In contrast to other composites, the composite PMCoG-1 and PMCoG-2 displayed nearly identical capacitive behaviour, as seen in Figure 11b. Based on the Rct and ESR value, the copolymer composite PMCoG-1 showed better electrochemical behavior than other composites. However, both CV and GCD favor the PMCoG-2 as better electrode material for electrochemical energy storage.

The present work is compared with previous literature for PANI, GO, and Co_3_O_4_ based electrode materials for energy storage applications Table 3.

## 4. Conclusions

The present work aimed to reinforce the poly (aniline-co-melamine) conductive matrix with GO sheets and Co_3_O_4_ nanoparticles for synthesizing ternary composites as an electrode material for electrochemical energy storage devices through simple co-precipitation and in-situ oxidative polymerization methods. The study is designed to assess the impacts of varying concentrations of GO with respect to its counterparts on the electrochemical aspects of composites. The characterizations of the synthesized materials were evaluated by the use of SEM, EDX, XRD, TGA, and FTIR. The electrochemical behavior of the electrode material was evaluated by using CV, GCD, and EIS. The composites displayed better electrochemical performance than controlled and binary composites. The copolymer composite PMCoG-1, PMCoG-2, and PMCoG-3 displayed a specific capacity of 100.63, 134.36, and 118.4 C/g, respectively. The composite PMCoG-2 (3 phr Co_3_O_4_ + 6 phr GO) exhibited the highest specific capacity due to synergy among the composite components. It is observed that as the concentration of GO was increased from 3 phr to 6 phr, the electrochemical performance of the electrode was improved. However, when GO concentration was raised to 12 phr, a decrease in the electrochemical active sites at the electrode deteriorated the electrochemical performance due to the heaping of the GO sheets. It is concluded that GO concentration up to 6 phr has a good impact on the electrochemical properties of the electrode. The composite PMCoG-2 is a better electrode material for energy storage applications.

## Figures and Tables

**Table 1:** Composition of binary and ternary composites of copolymer/GO/Co_3_O_4_.

SNo	PANI	Melamine	Graphene Oxide	Co_3_O_4_	Formulation Code
1	pure	-	-	-	PANI
2	-	pure	-	-	MA
3	-	-	pure	-	GO
4	-	-	-	pure	Co_3_O_4_
5	100	6 phr	-	-	PM (copolymer)
6	100	6 phr	-	6 phr	PMCo
7	100	6 phr	3 phr	3 phr	PMCoG-1
8	100	6 phr	6 phr	3 phr	PMGCoG-2
9	100	6 phr	12 phr	3 phr	PMGCoG-3

phr—part per hundred part of resin.

**Table 2:** Showing the weight losses in pristine and composites in a temperature range of 35–800 °C.

SNo	Formulation	Temp Range	Ist Weight Loss %	Temp Range	2nd Weight Loss %	Temp Range	3rd Weight Loss %	Total Weight Loss %	Residue Left
1	PANI	30–150 °C	11.62	150–360 °C	9.52	360–784	77.63	98.77	1.23
2	Melamine	30–150 °C	No loss	150–360 °C	56.09	360–782	41.99	98.08	1.92
3	GO	30–150 °C	15.29	150–360 °C	73.69	360–782	14.54	88.23	11.77
4	Co_3_O_4_	30–150 °C	4.22	150–360 °C	1.5	360–781	0.83	6.55	93.45
5	PM (copolymer)	30–150 °C	9.68	150–360 °C	11.78	360–785	54.41	75.87	24.13
6	PMCoG-1	30–150 °C	8.76	150–360 °C	15.3	360–785	64.91	88.97	11.03
7	PMCoG-3	30–150 °C	10.61	150–360 °C	9.72	360–782	35.53	55.86	44.14

**Table 3:** Comparison of present work with previous literature for PANI, Co_3_O_4_, and GO-based electrode material for energy storage application.

Electrode Material	Specific Capacity	Electrolyte	Reference
PANI@CNT/MnO_2_	143.26 C/g at 3 mV/s	1 M KOH	[7]
PANI/Cobalt intercalated metal organic framework (MOF)	154 C/g	1 M KOH	[68]
Ag/Co_3_O_4_@PANI ternary composite	262.62 C/g at 3 mV/s	0.1 M KOH	[78]
Co_3_O_4_ nanoflake	108.8 C/g at 3 mV/s.	1 M KOH	[4]
rGO-Co_3_O_4_-Ag	94.20 C/g	1 M KOH	[9]
Strontium phosphide-polyaniline	191.8 C/g at 3 mV/s	1 M KOH	[79]
Poly(aniline-co-melamine)/GO/Co_3_O_4_	134.36 C/g at 3 mV/s	1 M KOH	Present work

## Data Availability

Data is contained within the article.
